# Increased DJ-1 and α-Synuclein in Plasma Neural-Derived Exosomes as Potential Markers for Parkinson’s Disease

**DOI:** 10.3389/fnagi.2018.00438

**Published:** 2019-01-14

**Authors:** Zhen-Hua Zhao, Zhi-Ting Chen, Rui-Ling Zhou, Xu Zhang, Qin-Yong Ye, Yin-Zhou Wang

**Affiliations:** ^1^Department of Neurology, Fujian Provincial Hospital, Provincial Clinical College of Fujian Medical University, Fuzhou, China; ^2^Department of Neurology, Fujian Medical University Union Hospital, Fuzhou, China

**Keywords:** α-synuclein, DJ-1, biomarker, plasma, exosome, Parkinson disease

## Abstract

The diagnosis of PD might be in difficulty, especially in the early stages. Therefore, the identification of novel biomarkers is imperative for the diagnosis and monitoring disease progression in PD. DJ-1 and α-synuclein, are two proteins that are critically involved in the pathogenesis of PD, and they have been examined as disease biomarkers in studies. However, no study exists regarding DJ-1 in plasma neural-derived exosomes. In the present study, the levels of DJ-1 and α-synuclein in plasma neural-derived exosomes were studied together in order to investigate novel biomarkers for PD. DJ-1 and α-synuclein in plasma and plasma neural-derived exosomes of the patients with PD and controls were quantified by ELISAs. The data revealed that the levels of DJ-1 and α-synuclein in plasma neural-derived exosomes and the ratio of plasma neural-derived exosomal DJ-1 to total DJ-1 were significantly higher in patients with PD, compared with controls, while levels of the two proteins in plasma exhibited no difference between the patients with PD and controls. However, no relationship was identified between biomarkers and disease progression. In addition, significant positive correlations between DJ-1 and α-synuclein in plasma neural-derived exosomes were found in the patients with PD and in healthy individuals. We hypothesize that DJ-1 in plasma neural-derived exosomes may be used as a potential biomarker as α-synuclein in PD and they might participate in the mechanism of PD together.

## Introduction

Parkinson’s disease (PD), characterized by a set of extrapyramidal motor features, is the second most common neurodegenerative disease worldwide. The beginning of disease-specific pathology occurs before the onset of classical clinical manifestations, and the symptoms of PD are not apparent until almost 60% of the dopamine neurons have died (Berardelli et al., 2013). PD in the early stages is prone to be misdiagnosed, due to a lack of suitable disease biomarkers and overt clinical symptoms. Consequently, there is still a substantial misdiagnosis rate.

Gene mutations of α-synuclein culminate in familial PD and α-synuclein has a close association with the pathogenic mechanism of heredofamilial and sporadic PD (Lucking and Brice, 2000; Braak and Del Tredici, 2017). DJ-1 is another gene product that has previously been associated with both heredofamilial and sporadic PD, and appears to be associated with oxidative stress, which is an acritical process related to PD (Choi et al., 2006). Consequently, DJ-1 and α-synuclein have been considered to be leading biomarkers for PD (Hong et al., 2010). However, it is presently unknown whether the combination of DJ-1 and α-synuclein will be a more suitable combination for the diagnosis of PD.

Cerebrospinal fluid (CSF) has been reported to function as an accurate and reliable source for biomarkers in PD (Farotti et al., 2017). However, obtaining CSF is a cumbersome process compared to blood sampling in a regular clinical setting. Blood is an accessible source for application in a clinical setting (Mehta and Adler, 2016). However, a principal disadvantage of blood biomarkers is that they do not directly reflect the conditions in the central nerve system and are more easily affected by the peripheral environment.

Exosomes containing proteins and other constituents of their cellular origin are a means of communication between cells. Exosomes might be a pathway for neurons to divert proteins from neurons into the CSF or into the peripheral blood via blood–brain barrier. These materials associated with PD, including α-synuclein, maybe excessive and/or in a toxic or insoluble structure. To confirm this hypothesis, Shi and colleagues injected mice intracerebroventricularly with 125I-labeled α-synuclein or tau. And, they observed that 125I-labeled α-synuclein or tau could be detected in neural-derived blood exosomes which expressed the neuronal adhesion molecule L1CAM (Shi et al., 2014, 2016). Exosomal α-synuclein in neural-derived blood exosomes was increased in patients with PD (Shi et al., 2014). However, to the best of our knowledge, there are no relevant studies on exosomal DJ-1 in neural-derived blood exosomes.

In the present study, a combination of chemical and immunochemical methods were used to harvest and enrich neural-derived exosomes from small volumes of plasma in quantities that was enough to detect the implicated proteins in the pathogenic mechanism of PD. The levels of DJ-1 and α-synuclein in plasma neural-derived exosomes were analyzed in order to evaluate any potential associations with PD, and to assess their suitability as biomarkers for the disease. In addition, the levels of α-synuclein in plasma neural-derived exosomes were analyzed to investigate any associations between DJ-1 and α-synuclein, and the relationship between these proteins and the progression of PD were further investigated.

## Materials and Methods

### Participants

This study was performed in the Department of Neurology of Fujian Provincial Hospital, Fuzhou, China, and was approved by the Ethics Committees of Fujian Provincial Hospital. Laboratorians and statistical analyst were blinded to any clinical information and the grouping. All individuals provided written informed consents.

From January 2016 to June 2017, a total of 39 PD patients were recruited. All patients with PD were diagnosed according to the United Kingdom Parkinson’s Disease Society Brain Bank criteria (Hughes et al., 1992). The healthy controls were the patients’ husbands or wifes, or healthy community volunteers. The individuals in the control group did not exhibit any signs or symptoms indicative of neurological disease. All individuals were excluded on the following criteria: the presence of (1) tumors, (2) essential tremors, secondary parkinsonism, or Parkinson-plus syndrome, (3) severe craniocerebral trauma, (4) inflammatory, infectious, or autoimmune diseases in the peripheral and central systems, (5) severe systemic diseases, such as anemia, hepatosis, heart failure, pulmonary disorders, and chronic renal failure, and (6) other neurodegenerative diseases.

All patients with PD and control subjects received an assessment which contained medical history, physical and neurological examinations, laboratory tests, and neuropsychological assessment. The laboratory evaluation consisted of routine blood parameters, serum electrolytes, blood urea nitrogen, creatinine, fasting blood glucose, vitamin B12 and thyroid stimulating hormone. The above results for all study participants were normal. All patients with PD were assessed using Hoehn-Yahr (H-Y) and Unified Parkinson’s Disease Rating Scale-III (UPDRS-III) scores. The duration of disease since the time of initial diagnosis was available and used for subsequent analysis. Blood samples were collected at fasting state in the morning, after patients and controls were enrolled in the present study, and all plasmas were stored at -80°C.

### Isolation of Plasma Neural-Derived Exosomes

Exosomes were isolated according to the experimental protocol described in a previous report (Goetzl et al., 2015). Briefly, 0.8 ml of plasma was incubated with 0.15 ml of thromboplastin-D (Thermo Fisher Scientific, Inc., Waltham, MA, United States) for 60 min. The plasma was then added to 0.35 ml of calcium- and magnesium-free Dulbecco’s balanced salt solution (DBS-2) with protease inhibitor cocktail (Roche Applied Science, Inc., Madison, WI, United States) and phosphatase inhibitor cocktail (Thermo Scientific, Inc.). Following centrifugation at 1,500 ×*g* for 20 min, the supernatant was mixed with 252 μl of ExoQuick exosome precipitation solution (EXOQ; System Biosciences, Inc., Palo Alto, CA, United States), and incubated for 1 h at 4°C. The resulting exosome suspensions were centrifuged at 1,500 ×*g* for 30 min at 4°C, and each precipitation was re-suspended in 150 μl of DBS-2 with inhibitor cocktails and 100 μl of 3% bovine serum albumin. The suspensions were subsequently incubated for 1 h at 4°C with 1 μg of mouse anti-human CD171 (L1CAM neural adhesion protein) biotinylated antibody (clone 5G3, eBioscience; Thermo Fisher Scientific, Inc.) and 25 μl of streptavidin-agarose resin (Thermo Scientific, Inc.) with 50 μl of 3% BSA. Following being centrifuged at 200 × g for 10 min at 4°C and removal of the supernatant, each precipitation was suspended in 50 μl of 0.05 M glycine-HCl (pH 3.0) by vortexing for 10 s. Subsequently, 0.45 ml of DBS-2 with 2 g/100 ml of BSA, 0.10% Tween 20 and the inhibitor cocktails, was added to each suspension. After vortex-mixing, each suspension was incubated for 10 min at 37°C with. Samples were then stored at -80°C prior to ELISAs. The protein concentration of exosomes from the neural source was determined using the Bradford protein assay (Tiangen Biotech, Co., Ltd., Beijing, China), with bovine serum albumin as a standard.

### Transmission Electron Microscopy

The exosomes were placed in a droplet of 2.5% glutaraldehyde in PBS buffer at pH 7.2 and fixed for 2 h at 4°C. The samples were washed in 0.1 M PBS buffer and fixed in 1% osmium tetroxide for 2 h at 4°C. The samples were then dehydrated for 10 min in increasing concentrations of alcohol (50, 70, 90, and 100%, repeated three times), then infiltrated with alcohol/propylene oxide (1:1) and propylene oxide for 10 min at room temperature, respectively. The samples were embedded in Quetol-812 epoxy resin and polymerized at 40°C for 2 h, 60°C for 4 h, and 80°C for 10 h. Ultrathin sections (100 nm) were cut using a Leica UC6 ultra-microtome, and then were stained with uranyl acetate for 15 min and with lead citrate for 10 min at room temperature. The results were observed in a FEI TecnaiSPIRIT transmission electron microscope (Camcor, Eugene, OR, United States), operated at 120 kV.

### ELISA Quantification of Exosome Proteins and Plasma Protein

The proteins in plasma neural-derived exosomes were quantified by ELISA kits for α-synuclein and human DJ-1 (R&D Systems, Inc., Minneapolis, MN, United States), according to the manufacturer’s protocol. Plasma α-synuclein and human DJ-1 were also quantified, and the test for biomarker was repeated 3 times.

### Data Analyses

In the present study, SPSS Statistics 19.0 (IBM, Corp., Armonk, NY, United States) was used for statistical analyses. *p* < 0.05 was accepted to be statistically significant in all cases. All continuous variables, including age, UPDRS-III scores, duration of disease, the levels of DJ-1 in exosomes, total DJ-1 in plasma, α-synuclein in exosomes, total α-synuclein in plasma, and their ratio are presented as the mean ± standard deviation (SD). A χ^2^ test was used for comparing the differences between sexes. In order to compare the differences between age and biomarkers between the groups, a *t*-test was used to detect statistically significant differences when the data were normally distributed, and a Mann–Whitney *U*-test when the data were not normally distributed. In order to evaluate correlations among the biomarkers, Pearson’s correlation coefficients for DJ-1 and α-synuclein of plasma were obtained. Spearman’s correlation coefficients were also obtained to evaluate the correlations among the biomarkers of which the data do not conform to the normal distribution. Receiver operating characteristic (ROC) curves for the biomarkers were generated to evaluate their sensitivities and specificities in distinguishing PD from healthy controls. The “optimum” cut-off value for a ROC curve was the point associated with the maximal sum of sensitivity and specificity. ROC curves for the combination of DJ-1 and α-synuclein were also calculated using logistic regression analysis.

## Results

### Evaluation of DJ-1 and/or α-Synuclein in Plasma and Plasma Neural-Derived Exosome as Biomarkers for PD

**FIGURE 1:**
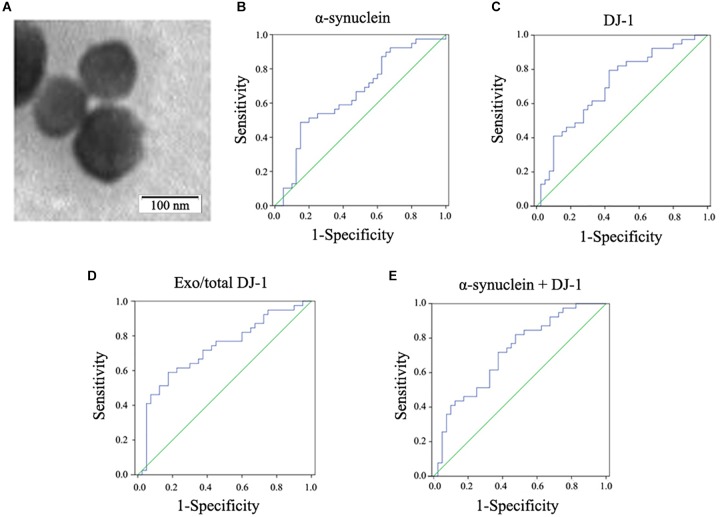
Observation of plasma neural-derived exosomes and ROC analysis of biomarkers for PD diagnosis. Exosomes with lipid bilayer structure were observed under the transmission electron microscope (black arrow). Scale bars: 100 nm **(A).** In the whole cohort, α-synuclein in plasma neural-derived exosomes provided an AUC of 0.654 (sensitivity = 48.7%, specificity = 85.0%) for PD versus controls **(B)**. DJ-1 in plasma neural-derived exosomes performed similarly (AUC = 0.703, sensitivity = 79.5%, specificity = 57.5%) in the whole cohort **(C)**. The AUC of the ratio of plasma neural-derived exosomal DJ-1 to total DJ-1 was 0.724 with a sensitivity of 59.0% and a specificity of 82.5% **(D).** The AUC of α-synuclein +DJ-1 was 0.714 **(E)**.

Plasma neural-derived exosomes were extracted and corroborated by transmission electron microscopy (Figure 1A). The concentrations of DJ-1 in the plasma neural-derived exosomes and plasma were measured using ELISAs. There was no significant difference in DJ-1 in the total plasma between patients with PD and controls in the total plasma (2.26 ± 0.65 vs. 2.49 ± 0.60 ng/ml, *p* = 0.104). The concentrations of DJ-1 in the plasma neural-derived exosomes were significantly higher in patients with PD compared with healthy controls (2.94 ± 0.96 vs. 2.34 ± 0.86 ng/mg, *p* = 0.002). The ratio of plasma neural-derived exosomal DJ-1 to total DJ-1 (exo/total) was also significantly higher in patients with PD, compared with the controls (1.39 ± 0.57 vs. 1.00 ± 0.50, *p* = 0.001) (Table 1).

**Table 1:** Compare of demographics and biomarkers between control subjects and PD.

		Parkinson’s disease stage				
	Control	Early	Advanced	Total	*P*1	*P*2
Number of cases	40	22	17	39		
Sex (M/F)	17/23	15/7	8/9	23/16	0.143	0.184
Age (years)						
Mean ± SD	66.6 ± 8.8	65.2 ± 11.2	67.5 ± 6.8	67.5 ± 6.9	0.820^b^	0.445^b^
Range	46–80	39–79	58–82	39–82	–	–
Duration of disease (years)						
Mean ± SD	–	3.9 ± 2.5	6.4 ± 3.6	5.0 ± 3.2	–	0.016^a^*
Range	–	0.3–9	2–15	0.3–15	–	–
UPDRS-III scores	–	37.8 ± 15.2	62.6 ± 19.3	48.6 ± 21.0	–	<0.001^b^*
Exosomes DJ-1 (ng/mg)	2.34 ± 0.86	2.97 ± 0.97	2.90 ± 0.97	2.94 ± 0.96	0.002^a^*	0.734^a^
Plasma DJ-1 (ng/ml)	2.49 ± 0.60	2.17 ± 0.51	2.39 ± 0.97	2.26 ± 0.65	0.104^b^	0.297^b^
Exo/total DJ-1	1.00 ± 0.50	1.44 ± 0.58	1.33 ± 0.58	1.39 ± 0.57	0.001^a^*	0.515^a^*
Exosomes α-syn (ng/mg)	6.50 ± 1.17	7.97 ± 2.97	7.46 ± 2.46	7.75 ± 2.74	0.018^a^*	0.650^a^
Plasma α-syn (ng/ml)	3.01 ± 1.17	3.04 ± 1.59	2.98 ± 1.44	3.01 ± 1.51	0.565^a^	0.820^a^
Exo/total α-syn	2.51 ± 1.45	3.19 ± 1.94	3.00 ± 1.68	3.11 ± 1.81	0.141^a^	0.777^a^



*exo, exosomes; α-syn, α-synuclein; ^a^Mann–Whitney U-test; ^b^Student’s t-test; P1, compare between control subjects and PD; P2, compare between early stage PD and advanced stage PD. ^∗^p < 0.05.*

The concentrations of α-synuclein in plasma neural-derived exosome and in plasma were also measured using ELISAs. Similar to the findings of a previous study (Shi et al., 2014), there was no significant difference in the plasma α-synuclein concentrations between patients with PD and controls (3.01 ± 1.51 vs. 3.01 ± 1.17 ng/ml, *p* = 0.565). However, the concentrations of α-synuclein in plasma neural-derived exosomes were significantly higher in patients with PD compared with healthy controls (7.75 ± 2.74 vs. 6.50 ± 1.17 ng/mg, *p* = 0.018 < 0.05) (Table 1).

To further evaluate the potential for DJ-1 and α-synuclein in plasma neural-derived exosomes to aid in the diagnosis of PD, ROC analysis was performed to characterize its sensitivity and specificity. The AUC for α-synuclein was 0.654, when the cut off value was 8.14 ng/mg, with a sensitivity of 48.7% and specificity of 85.0% (Figure 1B). The ROC analysis performance of DJ-1 in plasma neural-derived exosomes was identified to be moderate (AUC = 0.703, sensitivity = 79.5%, specificity = 57.5%) (Figure 1C), as the cut off values was 2.06 ng/mg. The exo/total DJ-1 generated a similar AUC result (0.724) with a sensitivity of 59.0% and a specificity of 82.5% at a cutoff value of 0.415 (Figure 1D). The AUC for the combination of DJ-1 and α-synuclein was 0.714 (Figure 1E), with a sensitivity of 82.1% and a specificity of 52.5% at a cutoff value of 0.35 on the predicted risk algorithm. The combination of these two proteins did not perform a significant discrimination.

### Evaluating the Relationships Between DJ-1 and α-Synuclein Levels of Plasma and Plasma Neural-Derived Exosomes

**FIGURE 2:**
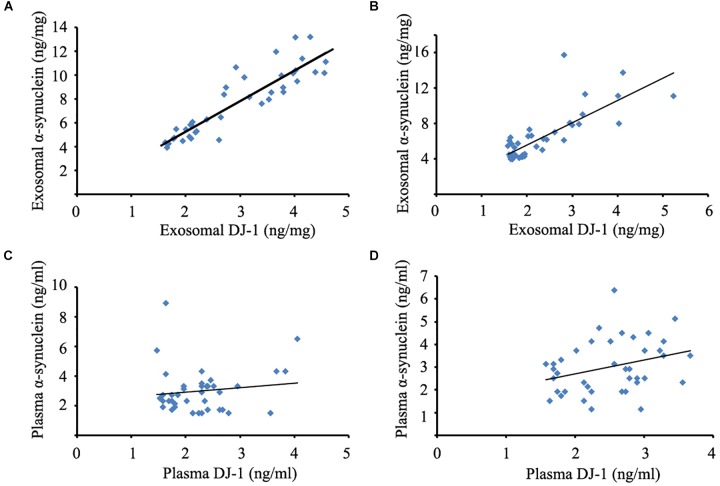
Correlation analysis between DJ-1 and α-synuclein of plasma and plasma neural-derived exosomes. A significant positive correlation between DJ-1 and α-synuclein of plasma neural-derived exosomes was observed in patients with PD (*rs* = 0.902, *p* < 0.001) **(A)**, as well as DJ-1 and α-synuclein of plasma neural-derived exosomes in controls (*rs* = 0.739, *p* < 0.001) **(B)**. No correlation between DJ-1 and α-synuclein of plasma was observed in patients with PD (*rs* = 0.059, *p* = 0.722) **(C)**. A weak correlation between DJ-1 and α-synuclein of plasma in controls (*rs* = 0.314, *p* = 0.048) **(D)**.

To evaluate the relationships between DJ-1 and α-synuclein levels of plasma and plasma neural-derived exosomes, the present study conducted a correlation analysis. A significant positive correlation was identified between DJ-1 and α-synuclein levels of plasma neural-derived exosomes in patients with PD (Figure 2A) and controls (Figure 2B). No correlation was found between the plasma levels of DJ-1 and α-synuclein in patients with PD (Figure 2C), but a weak correlation was found in controls (Figure 2D).

### Correlation Between DJ-1 or α-Synuclein and the Progression of PD

Patients with PD were classified into H-Y stages 1–5 (Hoehn and Yahr, 1967), and were divided into two groups, early stage PD (H-Y 1 to 2) which included 22 patients and advanced stage PD (H-Y 3 to 5) which included 17 patients. Patients with PD in the advanced stage had a longer disease duration and higher UPDRS-III scores when compared with patients with PD in the early stage. However, no significant differences were identified in the neural-derived exosome and plasma levels of DJ-1 and α-synuclein between patients with PD at the early stage and advanced stages of disease (Table 1).

## Discussion

The development of effective biomarkers is an important and urgent task for PD in the early stage. A reliable biomarker can be conductive to identifying patients with PD in the early stage. And, it can also be used for the development of new therapy to treat the disease.

Plasma neural-derived exosomes may serve as a potentially sources that accurately reflects the changes of the central nervous system (CNS) (Shi et al., 2016). Exosomes from CNS which contain jettisoned and potentially toxic forms of α-synuclein or other disease-associated proteins that may be transported into the systemic circulation system (Shi et al., 2014). In addition, these exosomes may avoid the interferences from the blood contamination, system inflammation, and potential tumor. The study of Fiandaca et al. (2015) revealed that the exosomal levels of P-S396-tau, P-T181-tau and Aβ1-42 were significantly higher than in controls at one to 10 years prior to diagnosis with Alzheimer’s disease. In a subsequent series of studies, astrocyte-derived exosomes or neural-derived exosomes were obtained from plasma by using the same method of polymer-based precipitation and combination of biotinylated antibody and streptavidin-agarose resin (Goetzl et al., 2015, 2016). In the present study, neural-derived exosomes were obtained using this method, and their origin was confirmed using transmission electron microscopy, and similar results were obtained when compared with a previous study (Shi et al., 2014). The principal advantage of this method using polymer-based precipitation, and combination of biotinylated antibody and streptavidin-agarose resin is that it is simple and can be easily performed in a typical laboratory without a Luminex workstation.

The α-synuclein in the CNS is associated with the progression of PD, and has been hypothesized to be secreted in means of exosomes (Marques and Outeiro, 2012; Gui et al., 2015). Previous studies have reported that plasma α-synuclein was not a suitable biomarker for PD (Lee et al., 2006; Li et al., 2007; Shi et al., 2010), as any peripheral cells containing abundant α-synuclein would affect the result (Barbour et al., 2008). In the present study, plasma α-synuclein levels exhibited no significant differences between patients with PD and controls. However, a higher level of α-synuclein was detected in plasma neural-derived exosomes in patients with PD by ELISA. These results were similar to those obtained by a previous study, in which the levels were detected by Luminex assays (Shi et al., 2014). As the results were free from methodological interference, the present study confirmed that α-synuclein in plasma neural-derived exosomes may function as a reliable biomarker for PD. However, there were limitations to the present study, namely that the sensitivity and specificity were not high enough. Therefore, the sample size needs to be expanded in future studies.

DJ-1 had previously been hypothesized to be associated with the mechanism of PD (Bonifati et al., 2003). Consequently, DJ-1 in biological fluids has garnered much interest as a promising biomarker for PD; however, the inconsistent results obtained by numerous studies regarding plasma DJ-1 suggest its unsuitability to be a reliable biomarker for PD (Waragai et al., 2007; An et al., 2018). Similar to α-synuclein, the plasmic level of DJ-1 is greatly affected by contamination due to its remarkably high content in erythrocytes (Shi et al., 2010). Therefore, researchers have focused on alternative sources of DJ-1. For example, a previous study reported that DJ-1 in saliva was increased in patients with PD compared with controls (Devic et al., 2011), and may present an association with disease progression (Kang et al., 2014). In a Korean study, the levels of DJ-1 in urinary exosomes in males were increased in patients with PD compared with controls and were increased with age in PD (Ho et al., 2014). Other forms of DJ-1, such as oxidized DJ-1 (Ogawa et al., 2014) and DJ-1 isoforms (Lin et al., 2012), have also been studied. To the best of our knowledge, the present study is the first to report that DJ-1 in plasma neural-derived exosomes was significantly increased in patients with PD compared with healthy controls. However, the combination of DJ-1 and α-synuclein in plasma neural-derived exosomes did not significantly enhance the performance of diagnosis.

In the present study, a significant positive correlation between DJ-1 and α-synuclein of plasma neural-derived exosomes was observed in patients with PD. The interaction of α-synuclein and DJ-1 serves an important role in heredofamilial and sporadic PD (Antipova and Bandopadhyay, 2017). A previous study reported that DJ-1 could be observed on the rim of Lewy bodies in patients with sporadic PD (Jin et al., 2005). The total level of DJ-1 protein is significantly increased in the brains of patients with PD compared with controls (Moore et al., 2005; Choi et al., 2006). In addition, DJ-1 can modulate the aggregation of α-synuclein and protect the neurons form cell toxicity of α-synuclein by chaperone-like activity (Sun et al., 2012) and physical interactions (Zondler et al., 2014). The knock-down or knock-out of DJ-1 has been demonstrated to increase α-synuclein toxicity, thus making neurons more vulnerable to the dopaminergic selective neurotoxin 6-hydroxydopamine (Batelli et al., 2015). The results of the present study further confirmed there is a correlation between DJ-1 and α-synuclein. Although the interactions between DJ-1 and α-synuclein have not been completely elucidated, DJ-1 and α-synuclein may participate together in the pathology of PD.

The mechanism for the increased α-synuclein and DJ-1 in the exosome is presently unclear. It has been hypothesized that α-synuclein may be secreted out of cell via exocytosis, exosomes, or other extracellular vesicles (Faure et al., 2006). In addition, there are several potential mechanisms for increasing α-synuclein in the exosome. Increasing α-synuclein synthesis may culminate in its increased secretion to the extracellular space in previous *in vitro* studies providing evidence for this potential mechanism. When neurons over-expressing α-synuclein would actively secrete an increased amount of α-synuclein into the extracellular space by the pathway of exosomes (Emmanouilidou et al., 2010; Reyes et al., 2015), α-synuclein induced cell death may be prevented by exosomal secretion. However, this self protective mechanism would damage neighboring cells (Lee et al., 2005). Another potential mechanism is the dysfunction of the autophagy-lysosomal pathway or macroautophagy. An example of this being ammonium chloride or bafilomycin A1, which inhibits lysosomal function, which would then increase the release of α-synuclein in exosomes (Alvarez-Erviti et al., 2011). This effect may also be observed in α-synuclein over-expressing SH-SY5Y cells treated with secretory carrier membrane protein 5, which is an autophagy inhibitor (Yang et al., 2017). If macroautophagy function was inhibited by silencing the key element autophagy related gene 5, the releasing of α-synuclein from cells by the pathway of exosomes would be observed as a kind of compensation (Fussi et al., 2018). The dysfunction of the endocytic pathway may be another mechanism for increasing α-synuclein secretion via the exosome. The glucocerebrosidase gene is a pathogenic gene in hereditary PD, and its mutation has been hypothesized to decrease glucocerebrosidase enzymatic activity and attenuate endocytic function. The inhibition of this enzymatic activity or expression of this mutant gene results in release of α-synuclein and its oligomers (Papadopoulos et al., 2018). Research regarding in exosomal DJ-1 is rare; however, we hypothesize that increasing DJ-1 in pathogenicity of α-synuclein maybe partially transferred with the exosome into extracellular space.

## Conclusion

α-Synuclein and DJ-1 in plasma neural-derived exosomes may serve as potential biomarkers for PD. However, further studies should be conducted with larger patient cohorts in order to corroborate the significance of these findings and the relationship of these biomarkers and disease progression.

## Author Contributions

Z-HZ was responsible for the conception and design of the present study, execution of the experimental work, and wrote the first draft of the manuscript, and review and critique of the Manuscript. Z-TC undertook study design, execution of statistical analysis, execution of experimental work, and the review and critique of the manuscript. R-LZ and XZ organized the research project and reviewed and critiqued the manuscript. Y-ZW organized the research project and review and critiqued the statistical analysis and the manuscript. Q-YY was responsible for the conception of experiments, execution of experimental work, design and execution of statistical analysis, and the review and critique of the manuscript.

## Conflict of Interest Statement

The authors declare that the research was conducted in the absence of any commercial or financial relationships that could be construed as a potential conflict of interest.
